# Observational studies: goldmines of information on rare diseases

**DOI:** 10.1186/s12916-017-0868-7

**Published:** 2017-05-12

**Authors:** Robert S. Benjamin

**Affiliations:** Department of Sarcoma Medical Oncology, The University of Texas MD Anderson Cancer Center, 1515 Holcombe Boulevard – Unit 450, Houston, TX 77030 USA

**Keywords:** METASARC, Retrospective review, Evidence-based medicine, Soft-tissue sarcoma, Combination chemotherapy, Local therapy of metastatic disease

## Abstract

The article by Savina et al. from the large METASARC database of the French Sarcoma Group (*BMC Med* 15:78, 2017) provides a wealth of information about the natural history and therapy of patients with metastatic soft tissue sarcomas. The information complements – and in some cases surpasses – that obtained from randomized clinical trials, and should not be overlooked because of its retrospective nature. For rare diseases, retrospective data are often more important than data from randomized trials because of the inherent restrictions on sample size. The article provides clear information regarding the different behaviors of different histological types of sarcoma, the importance of localized therapy for metastatic disease, and the critical role of combination chemotherapy in initial treatment to improve survival.

Please see related article: https://bmcmedicine.biomedcentral.com/articles/10.1186/s12916-017-0831-7

## Background

In recent years, medical education has emphasized the importance of evidence-based medicine [1]. Knowledge derived from randomized clinical trials (RCTs) and their critical appraisal has become a cornerstone in medical practice. Meanwhile, phase II studies, retrospective analyses and expert opinion are often considered ‘background information’ and afforded little weight in decision-making. Unfortunately, for those interested in rare diseases such as soft-tissue sarcomas (STS), or even rarer diseases, for example the 75 or so different diseases comprising STS, RCTs are scarce. Minor flaws in study design or bias in study entry make interpretation of results problematic [2]. The review of the METASARC database by Savina et al. [3] is informative to those who are open to non-RCT-based data and might even be considered a textbook on the natural history and treatment of these tumors.

## The METASARC study

The METASARC database collected information on treatment and outcomes of 2165 adult patients with metastatic STS, 1575 of whom received at least one systemic treatment. Patients were enrolled, or not, in clinical trials, and were treated by members of the French Sarcoma Group from 1990–2013. Only patients with usual STS histologies were included; those with gastrointestinal stromal tumors and Ewing sarcomas were excluded because their treatment is disease-specific and unlike that of other STS. Primary visceral sarcomas were also excluded. Care was coordinated by three national reference centers. Unlike some clinical trials that do not require expert pathology review prior to study entry, an expert sarcoma pathologist confirmed the diagnosis, grade, and subtype of each sarcoma before entering this data into the database.

## Alternative endpoints

Since many patients were not enrolled in formal clinical trials, time to next treatment (TNT) was used as an endpoint rather than time to progression (TTP, or progression-free survival, PFS, in a clinical trial). Multivariate analysis also revealed that patients had good overall survival (OS). While formal RECIST (response evaluation criteria in solid tumors) progression was not measured for patients not enrolled in clinical trials, caregivers’ practice was presumably to change therapies when disease progression was suspected. The authors recommend prospective evaluation of TNT as a clinical trial endpoint, but I doubt it will add to PFS for patients in formal trials, nor would it be a meaningful endpoint if practice were to change treatments after a fixed number of cycles or at maximum response.

## Histology-specific observations

Concordant with our own observations, patients with leiomyosarcomas had longer TNT and OS than those with other histologies. Perhaps this is why the EORTC phase II study of eribulin suggested activity against leiomyosarcoma [4], while the phase III study showed that this activity was confined to the liposarcomas [5]. Neither the active nor inactive control groups from the EORTC database accounted for the superior prognosis of the leiomyosarcoma subset in previously treated patients [6]. If TTP or PFS are to be used as benchmarks in future studies, disease-specific benchmarks are critical.

Unlike patients with leiomyosarcomas, patients with unclassified pleomorphic sarcomas (UPS) had a poor prognosis. This is the first study of a large cohort of patients with UPS in the age of modern immunohistochemistry, so patients with myogenic or lipogenic differentiation who might previously have been grouped together as having malignant fibrous histiocytomas (MFH), have been eliminated. Patients with UPS had among the shortest TNT and OS. Clearly, new therapies aimed at this patient subset need development. Notably, while gemcitabine is used in Europe, it has mostly been used in patients with leiomyosarcoma. In the SARC study of gemcitabine versus gemcitabine plus docetaxel, the best results were seen in patients diagnosed as having MFH [7]. It is impossible to judge whether those patients would now be diagnosed as having UPS, or whether they might have had sufficient myogenic differentiation to be diagnosed with leiomyosarcoma, but formal studies in UPS might be worthwhile. The investigators should consider this regimen for patients with UPS, if they are not already doing so.

## Combination chemotherapy improves survival

**Fig. 1:**
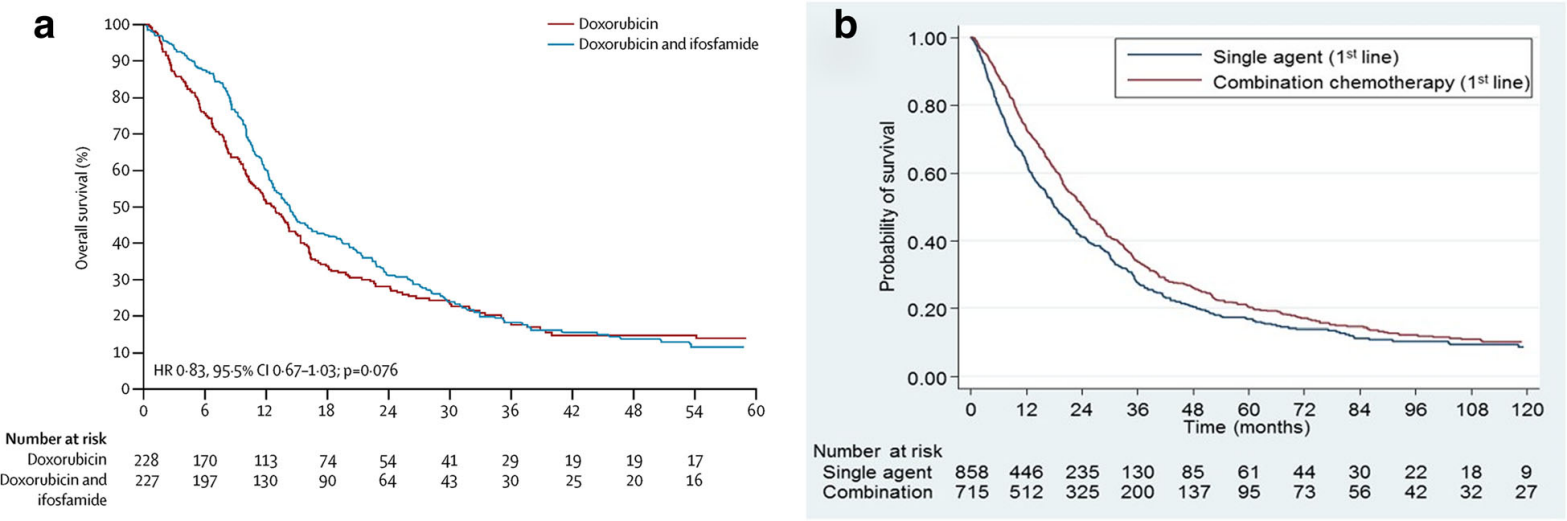
The shape of the survival curves and the hazard ratio are similar in both studies, indicating a clear survival advantage for combination chemotherapy over single-agent doxorubicin. **a**. Survival of patients with soft-tissue sarcomas receiving combination chemotherapy or single-agent doxorubicin. The hazard ratio was 0.83 (95% CI 0.67–1.03); *P* = 0.076. Reprinted from Judson et al. [8] (copyright 2017) with permission from the primary author and Elsevier. **b**. The hazard ratio was 0.822 (95% CI 0.724–0.932); *P* = 0.0003. Reprinted from Savina et al. [3]

An important observation from this study is the improved survival of patients treated with combination chemotherapy, usually doxorubicin and ifosfamide. The authors emphasize that combination chemotherapy is particularly indicated when tumor shrinkage is expected to provide clinical benefits, and warn about increased toxicity. In fact, those conclusions were reached in the excellent but underpowered RCT by Judson et al. in 2014 [8], and had previously been reached by Santoro et al. in 2005 [9]. However, multivariate analysis of data from the METASARC database showed that using combination chemotherapy as the first line of metastatic treatment conveyed a statistically significant benefit in OS (*P* = 0003). The hazard ratio for improvement was 0.822. The prospective RCT by Judson et al. showed an identical hazard ratio of 0.83, but was only powered (with 455 patients) to detect a maximum hazard ratio of 0.737. Therefore, the survival advantage demonstrated (*P* = 0.076) was discarded, and the authors concluded there was no survival advantage. At that time, we suggested there was a survival advantage [2], and the METASARC data strongly support that conclusion. Although the data are not derived from a prospective RCT, the survival curves from both the METASARC analysis and the Judson trial have the same shape and hazard ratio (Fig. 1). Data from the observational study confirm and add weight to the data from the prospective RCT, despite the fact that the trial reached the wrong conclusion because it was inadequately powered. I suggest that the observational study provides higher-level evidence than the RCT.

Patient selection might account for the superior survival seen by Savina and colleagues, since the best patients were chosen for combination chemotherapy. However, patients chosen to receive single agent therapy also had superior survival to those in the RCT. The relative benefit derived from the combination therapy is quantified by the hazard ratio, which was identical in both the Savina and Judson reports. It is time to stop recommending single-agent doxorubicin as initial therapy for any patient with metastatic STS who is fit enough to tolerate a combination, and direct future studies to improving the results.

## Localized therapy of metastatic disease

Patients who underwent additional localized therapy for metastatic disease did better than those who were treated only systemically. Patients who can be rendered disease-free by local or systemic therapy will live longer than those who are never disease-free. The data from this analysis support that observation because localized therapy is rarely attempted if it is unlikely to result in a disease-free (or total disease control) outcome. Localized strategies for metastatic disease are particularly suited to patients with sarcomas, because metastatic sites tend to be more limited than in other malignancies.

## Additional observations

In the patients studied, little value was demonstrated from third or subsequent lines of systemic therapy, except for patients with leiomyosarcoma. I have less faith in this observation than others. Many patients were studied before newer active agents like pazopanib or trabectedin were available. The gemcitabine–docetaxel combination is rarely used for tumors other than leiomyosarcoma, yet our own experience suggests that it should be used [7]. Prolonged benefit has been seen from therapy past second-line in patients with sarcomas other than leiomyosarcoma.

The advantage of being enrolled in a clinical trial has also been observed with other tumors. Only patients with good performance status and expected survival are eligible for trials, so they might be expected to do better. However, until recently, active drugs for the treatment of sarcomas were only available in clinical trials. Survival of these patients, of whom not all were enrolled in clinical trials, was substantially better than that reported from the front-line EORTC study [8].

A surprising number of patients were not treated with any systemic chemotherapy. The diagnosis of metastatic sarcoma may have been made too late to have any therapeutic impact. Underlying heart disease might have also made the treating physicians reticent to recommend doxorubicin therapy. However, we have safely used doxorubicin in elderly patients with coronary artery disease with continuous infusion [10] or with dexrazoxane [11]. If patients are monitored closely and cumulative dose limits are observed, they are unlikely to develop clinically significant cardiac toxicity.

## Conclusions

Savina et al. provide valuable information on the natural history and therapy of metastatic STS. Those interested in rare diseases should not ignore studies like this just because they are retrospective. The power of a large database, expert pathology review, and expert management should be welcomed, not ignored. As shown, the superior survival of patients treated with combination chemotherapy was almost identical in both this large, adequately powered retrospective study and the smaller, underpowered, prospective RCT, but the METASARC study reached the correct and opposite conclusion to the prospective trial. Let us not ignore the evidence just because we have been taught that it is in the wrong format. Perhaps our teachers never treated rare diseases.
